# Modified Fanning Technique of Biostimulant Delivery in Aesthetic Medicine

**DOI:** 10.1111/jocd.70086

**Published:** 2025-03-18

**Authors:** Anna Grodecka, Anna Piotrowska

**Affiliations:** ^1^ Medestetis Clinic Krakow Poland; ^2^ Department of Chemistry and Biochemistry, Institute of Basic Sciences, Faculty of Motor Rehabilitation University of Physical Culture in Krakow Krakow Poland

**Keywords:** biostimulants, calcium hydroxyapatite, cannula techniques, polycaprolactone, poly‐L‐lactic acid, skin aging

To the Editor,

1

Skin aging is a progressive degradation manifested primarily by structural weakening and loss of aesthetics through dulling, dehydration, and pigmentation changes. A number of methods are used to eliminate these age‐related changes. In aesthetic and bioregenerative treatments, so‐called biostimulants are used, among others. The basic purpose of their use is to improve the quality of the skin. The most important features associated with great popularity and frequency of use in patients are minimal invasiveness, safety of use, and short post‐treatment regeneration time. Biostimulants have become a cornerstone in aesthetic medicine, offering non‐invasive solutions to enhance skin rejuvenation, counteract aging and promote tissue regeneration. These agents leverage the body's natural healing and regenerative capacities to achieve cosmetic improvements.

As a practitioner with over 17 years of experience in aesthetic medicine, I have observed a potential limitation in the widely accepted use of cannula techniques for administering biostimulators. While cannulas have traditionally been favored for their safety and precision [1, 2], my clinical practice and theoretical analysis suggest that this method may inadvertently lead to suboptimal results due to uneven distribution of the product over tissue surfaces.

Using a cannula, an equal amount of biostimulator is deposited along its trajectory (Figure 1). However, due to the geometry of injection, the tissue surface area covered by the product near the cannula entry point is significantly smaller than that in more distal regions. This disparity can be mathematically illustrated using the area of quarter circles, where the surface area near the entry point (35 mm radius) is approximately 962 mm^2^, while the distal area between 35 mm and 70 mm radii is 2886 mm^2^—three times larger. Consequently, the distal tissue receives significantly less product per unit area.

**FIGURE 1:**
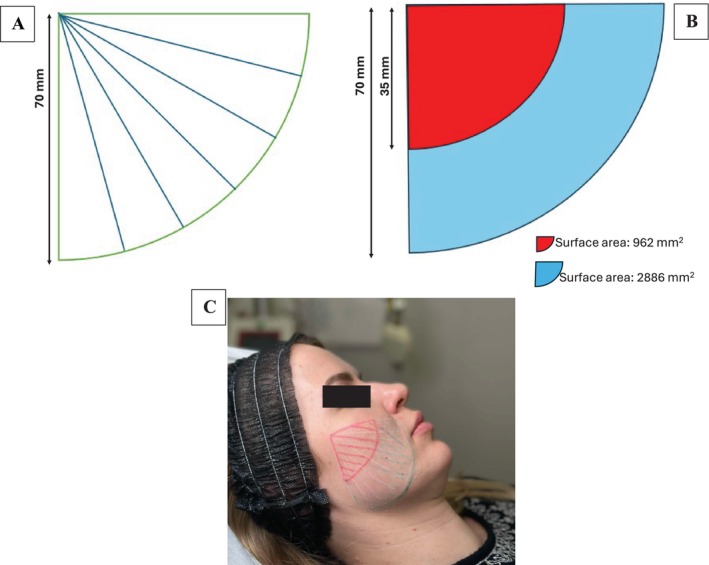
Graphic presentation of the skin area treated with the cannula (70 mm). (A) The fan technique used today; (B) Areas of sections of the full cannula radius and half the cannula length; (C) Photographic presentation.

2

The efficacy of biostimulators, such as poly‐L‐lactic acid, calcium hydroxyapatite and polycaprolactone, depends on their interaction with tissue to induce neocollagenesis and other regenerative processes [3, 4]. Uneven distribution may compromise the efficacy of stimulation in distal regions, where the lower concentration of product molecules per tissue unit area could result in suboptimal collagen induction. This issue is particularly relevant given that biostimulator effectiveness is highly dependent on sufficient molecular interaction with targeted tissues [5].

While this effect may be negligible for hyaluronic acid fillers, where volume and contouring are primary goals, it becomes critical for biostimulators, where tissue regeneration and remodeling are the primary mechanisms of action. Studies have highlighted the importance of uniform distribution to optimize outcomes with biostimulators [6].

To address this issue, we propose a revised injection protocol aimed at achieving more uniform tissue coverage (Figure 2). In this protocol, the product administration in distal regions will be doubled to compensate for the increased tissue surface area. This adjustment will be achieved by modifying the injection pattern: instead of depositing the product continuously along the entire length of the cannula, every other pass will terminate halfway along the cannula's trajectory from the distal end. This selective deposition method ensures a higher concentration of biostimulator in distal regions, where it is needed most.

**FIGURE 2:**
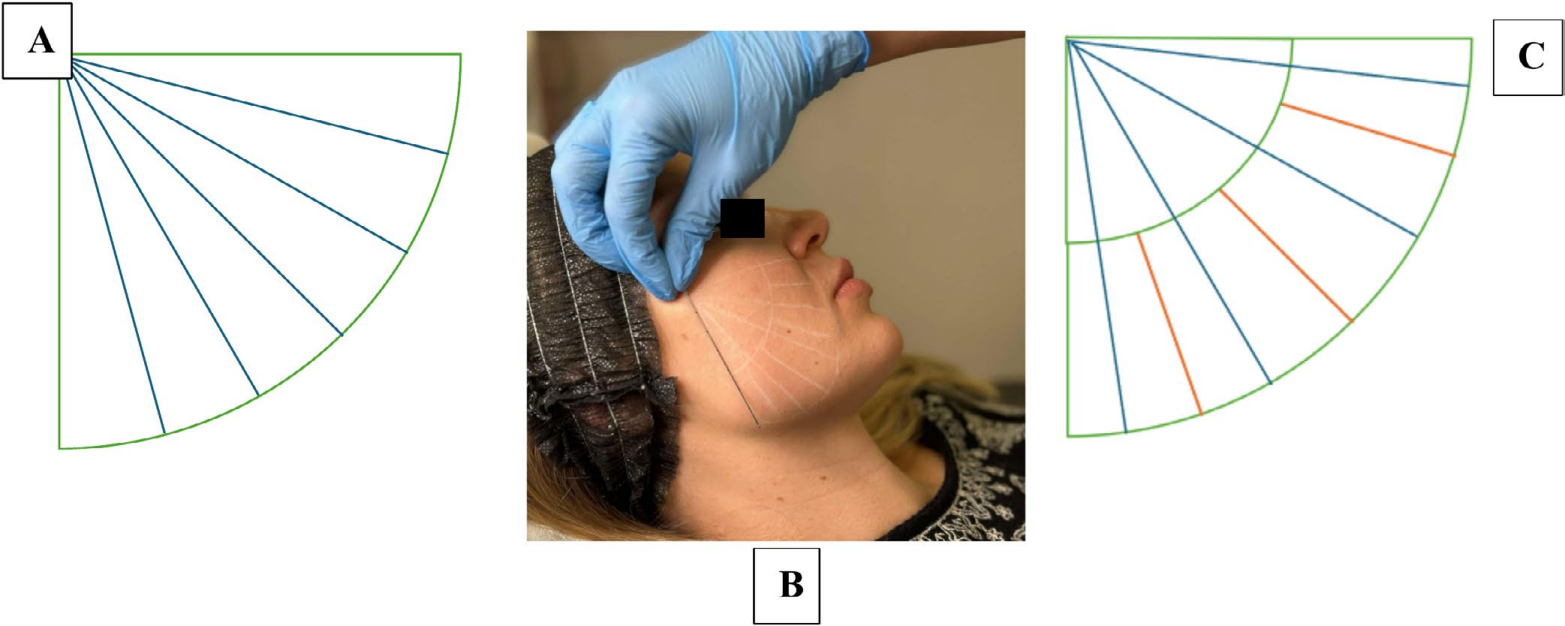
Proposal for modification of the fanning technique using a cannula (70 mm). (A) The fan technique used today; (B) Modified technique—photographic presentation; (C) Diagram of the modified technique (alternating sections of full radius and half radius lengths).

3

To our knowledge, the modification of the fanning technique shown here is proposed for the first time. Further clinical trials and imaging studies are necessary to validate the effectiveness of this protocol in achieving optimal outcomes. By tailoring injection techniques to the specific requirements of biostimulators, we can potentially improve their therapeutic efficacy and provide better results for patients.

## Ethics Statement

This research did not require Institutional Review Board approval because it did not involve any procedures requiring Bioethics Committee approval. The model signed permission to use her photos in the publication.

## Conflicts of Interest

The authors declare no conflicts of interest.

## Data Availability

Data sharing not applicable to this article as no datasets were generated or analysed during the current study.
